# Principal component analysis of MALDI TOF MS mass spectra separates *M. abscessus (sensu stricto)* from *M. massiliense* isolates

**DOI:** 10.1186/s12866-016-0636-4

**Published:** 2016-03-01

**Authors:** Jan Kehrmann, Sarah Wessel, Roshni Murali, Annegret Hampel, Franz-Christoph Bange, Jan Buer, Frank Mosel

**Affiliations:** Institute of Medical Microbiology, University Hospital Essen, University Duisburg-Essen, Essen, Germany; Department of Medical Microbiology and Hospital Epidemiology, Hannover Medical School, Hanover, Germany

**Keywords:** MALDI-TOF MS, VITEK MS, Mycobacteria, Identification, Nontuberculous mycobacterium

## Abstract

**Background:**

The discrimination of the members of the *Mycobacterium abscessus* complex is of clinical interest because one of the subspecies, *M. massiliense,* exhibits higher rates of response to antibiotic treatment for lung infection than do the other members of that complex. *M. abscessus* complex contains three subspecies that are laborious to identify; therefore, a routine diagnostic tool would be worthwhile.

**Results:**

We used principal component analysis, hierarchical cluster analysis, and single-peak analysis to examine peak lists derived from matrix-assisted laser desorption/ionization time-of-flight mass spectrometry (MALDI TOF MS) mass spectra of 50 clinical *M. abscessus* complex isolates, including 28 *M. abscessus (sensu stricto)*, 19 *M. massiliense,* and 3 *M. bolletii* isolates grown in mycobacterium growth indicator tube liquid medium and prepared with a bead-based protocol. Principal component analysis but not hierarchical cluster analysis separated *M. abscessus (sensu stricto)* isolates and *M. massiliense* isolates into two clusters. Furthermore, single-peak analysis displayed 4 discriminating peaks that separated *M. abscessus (sensu stricto)* from *M. massiliense* isolates. *M. bolletii* isolates did not exhibit specific peaks but resembled the *M. abscessus (sensu stricto)* peak profile and also grouped within this principal component analysis cluster. Principal component analysis of all peak lists with the exclusion of the four discriminating peaks again separated *M. abscessus (sensu stricto)* from *M. massiliense* isolates, thus relativizing the importance of these peaks for subspecies identification.

**Conclusions:**

Principal component analysis of peak lists derived from MALDI TOF mass spectra is a robust and convenient method of discriminating *M. massiliense* isolates from the other members of the *M. abscessus* complex.

## Background

*Mycobacterium abscessus* is a rapidly growing mycobacterium that is involved in soft-tissue and bone infections [1]. It is the most frequently isolated nontuberculous mycobacterium (NTM) found in European cystic fibrosis (CF) patients and has emerged as an important pathogen in CF lung disease [2, 3]. *M. abscessus* complex is split into three subspecies, *M. abscessus (sensu stricto)*, *M. massiliense,* and *M. bolletii,* which are commonly separated by multilocus analysis of hsp65, rpoB, secA, and the 16-23S internal transcribed spacer (ITS) region [4, 5]. However, the nomenclature is still being debated; a recent proposal suggests uniting *M. massiliense* and *M. bolletii* to form *M. abscessus* subsp*. bolettii comb. nov*. and to distinguish that new combination from *M. abscessus* subsp. *abscessus* [6].

*M. abscessus* lung disease is difficult to treat, and the subspecies differ in antibiotic resistance [7]. The presence of an inducible methylase gene [erm (41)] in *M. abscessus (sensu stricto)* results in macrolide resistance under clarithromycin regimen therapy, while a functional *erm(41)* gene is missing in *M. massiliense*. This inducible resistance to clarithromycin is considered to explain the low efficiency of macrolide-containing therapy regimens in *M. abscessus (sensu stricto)* disease in contrast to the high treatment response rates in *M. massiliense* disease [8–10]. Furthermore, a rare mutation within the *rrl* gene was reported to be responsible for macrolide resistance in *M. abscessus* complex [11]. Macrolides are the major therapeutic agents for *M. massiliense*, but the importance for *M. abscessus (sensu stricto)* is less clear. Many experts recommend a combination of parenteral agents based on in vitro MICs for macrolide resistant *M. abscessus* complex strains with amikacin and cefoxitin [10]. Due to the varying antibiotic therapy response of the different members of the *M. abscessus* complex, subspecies identification is of particular clinical interest. As their identification by using molecular methods and sequencing of several genes is cumbersome and not available in many laboratories, a routine diagnostic tool is desirable.

Several studies have used matrix-assisted laser desorption/ionization time-of-flight mass spectrometry (MALDI-TOF MS) to identify mycobacteria. However, this method currently cannot reliably differentiate the subspecies of *M. abscessus* complex [12–14]. The current versions of the Vitek MS databases (bioMérieux, Durham, NC, USA), Saramis v.4.12 (Vitek MS Plus, research use only [RUO]) and in vitro diagnostic (IVD 2.0), identify isolates at the level of the *M. abscessus* complex but do not separate *M. abscessus (sensu stricto), M. massiliense,* and *M. bolletii*. In the present study we determined the performance of Vitek MS Plus in discriminating the various subspecies by using the peak lists of the MALDI TOF mass spectra for principal component analysis (PCA), hierarchical cluster analysis (HCA), and single-peak analysis.

## Methods

### Strains and cultures

The study was performed in accordance with the Declaration of Helsinki and was approved by the ethics committee of the Medical School Hannover (protocol number 3023-2016). 50 pseudo-anonymized *M. abscessus* complex clinical isolates from the Department of Medical Microbiology and Hospital Epidemiology of the Hannover Medical School were used in this study; these isolates had been isolated from patient samples as part of standard care, collected as part of epidemiological surveillance and were characterized previously [15]. No written informed consent was necessary for this type of study. The identification of *M. abscessus (sensu stricto)* (*n* = 28), *M. massiliense* (*n* = 19), and *M. bolletii* (*n* = 3) had been performed with sequencing of 16S rRNA and genotypic analysis of the *hsp65*, *erm(41)*, *rrl,* and *rrs* genes [15]. Isolates were grown from frozen stocks in mycobacterium growth indicator tube (MGIT, Becton, Dickinson and Company) liquid medium at 37 °C and were analyzed after 1 week of culture.

### Mycobacterial preparation protocol

The mycobacterial preparation protocol suggested by bioMérieux was used with few modifications. We pipetted a 1.8-ml aliquot of liquid medium from the bottom of the positive MGIT culture into a centrifuge vial and subjected it to centrifugation at 6,000 × g for 10 min. The supernatant was discarded, and the pellet was resuspended in 500 μl of 70 % ethanol. After centrifugation at 6,000 × g for 10 min, the pellet was resuspended in another 500 μl of 70 % ethanol and was transferred into a centrifuge vial containing 200 μl of glass beads (Carl Roth GmbH & Co., Karlsruhe, Germany), each 1 mm in diameter. The second ethanol washing step was added to the original preparation protocol of the manufacturer to improve removal of remnants. Furthermore, we used glass beads, 1 mm in diameter instead of 0.5 mm in diameter. The cells were disrupted with a vortex (MS3 digital shaker; IKA, Wilmington, NC, USA) for 15 min and then incubated at room temperature for 10 min. The suspension was transferred into a sterile tube and subjected to centrifugation at 18,000 × g for 2 min. The pellet was resuspended in 10 μl of 70 % formic acid. After the addition of 10 μl of 100 % acetonitrile, the suspension was briefly vortexed and subjected to centrifugation at 18,000 × g for 2 min. Next, 1 μl of the supernatant was pipetted onto the MALDI-TOF target slide. The samples were dried at room temperature, and 1 μl of α-cyano-4-hydroxy cinnamic acid (CHCA) matrix solution (bioMérieux, Marcy l’Etoile, France) was added. Samples were analyzed in quadruplicate after the matrix had crystallized at room temperature.

### Data processing

Lists of the relative peak intensities (range, 2,000 Da to 20,000 Da mass-to-charge ratio [m/z]) derived from the raw data were used as input for HCA and PCA. We used the presets recommended by bioMérieux for smoothing, baseline subtraction, and peak detection. To compensate for small differences in the m/z values of corresponding peaks across measurements that resulted from the limited accuracy of the measurement process itself and the calibration and peak-detection algorithm, we divided the recorded m/z range from 2,000 Da to 20,000 Da into 3,600 bins of 5-Da widths, which is close to the mass accuracy assumed by the database-matching algorithm (0.08 %).

HCA and PCA, two separate cluster analytical methods, were performed with DataLab software (Epina GmbH, Pressbaum, Austria). For HCA, Ward’s method was used to calculate the Euclidean distance after standardization. HCA generates clusters by simply pairing individuals or subclusters that are the smallest distance apart in the space, with the original parameters as the coordinates (in our case, m/z values). PCA is a type of multivariate analysis that reduces factors when there is redundancy in the data. It generates a new coordinate system by linear combination of the original coordinates (m/z-values), which represent the principal components (PCs). This coordinate transformation is performed in such a way that the origin moves into the center of mass and the new coordinate axes are rotated so that they are now collinear to the principal axes of the data cloud. The PCs, as the new coordinates, are numbered according to the percentage of variance in the original data that they explain. The first three of the orthogonal and therefore linearly independent new coordinates (PCs 1, 2, and 3) should preferably be used to visualize the data as dots in a 3-dimensional space.

We cannot expect that the redundancy of the original 3,600 parameters is so great that the first three PCs alone will explain most of the total variance. Even so, it is possible to reveal substructures appearing as clusters, even if the number of involved original parameters (m/z-values) that make up the difference is small, or if the differences themselves are small. PCA weights those parameters with a high loading factor, thereby amplifying their contribution to the new PCs to the disadvantage of parameters with only marginal variance. It is, simply speaking, this “amplification” that, in some settings, allows PCA to reveal reasonable clusters that are determined by small differences caused by the real grouping of individuals with predominantly similar parameters, something that HCA does not do. In such cases, using HCA is difficult because the differences in the Euclidean distances between individuals in the same group are comparable to those between individuals in other groups.

## Results

All isolates included in the study were correctly identified as *M. abscessus* complex by analysis with Vitek MS Plus RUO software, which is based on SuperSpectra. Of the 50 isolates, 47 were identified with the highest possible confidence level (c.l.) of 99.9 % (26 of 28 *M. abscessus (sensu stricto)* isolates, 18 of 19 *M. massiliense* isolates, and 3 of 3 *M. bolletii* isolates). The 3 remaining isolates were correctly identified with a c.l. of 94.5 %. Because the current versions of the VITEK MS databases (Saramis 4.12 (RUO) and IVD) cannot differentiate between the subspecies of the *M. abscesssus* complex (*M. abscessus (sensu stricto), M. massiliense,* and *M. bolletii),* we accessed the raw spectra and peak lists of all isolates by using the VITEK MS Plus RUO system to study unique differences between the subspecies by HCA, PCA, and single-peak analysis.

HCA using Ward’s method, a commonly used approach, created several large mixed clusters with subclusters containing 2 to 6 isolates of a subspecies each; however, it did not reproduce the grouping according to the subspecies (data not shown).

**Fig. 1:**
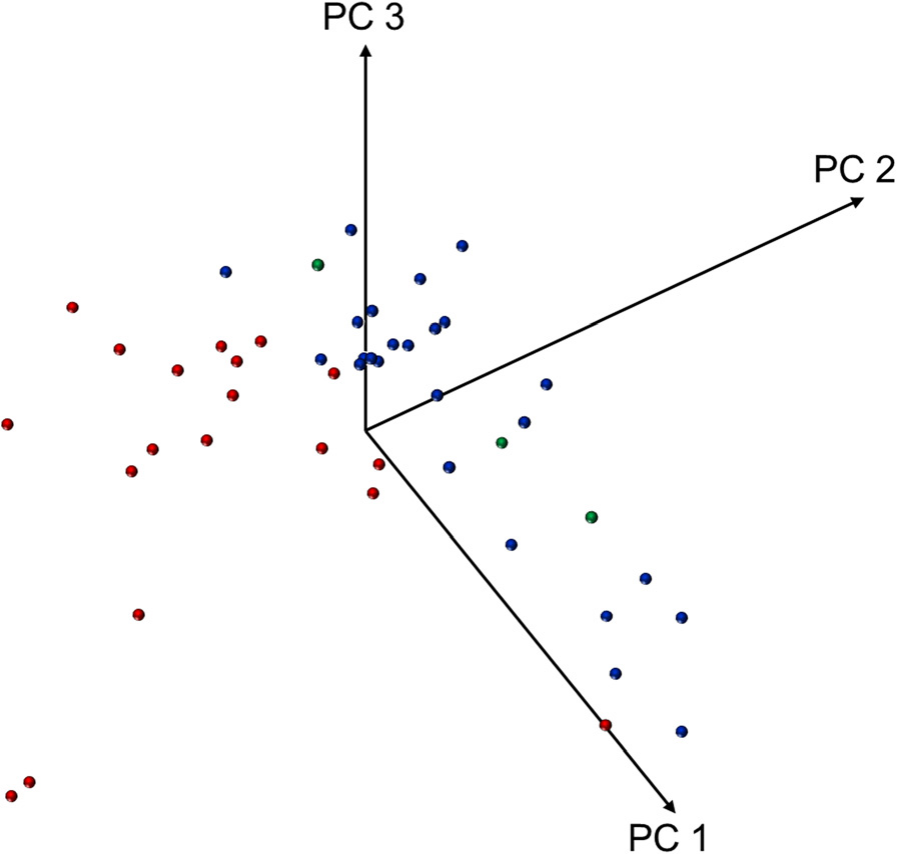
Principal component analysis of the entire matrix-assisted laser desorption/ionization time-of-flight mass spectrometry (MALDI TOF MS) peak lists of *Mycobacterium abscessus* complex isolates. Diagram of the first three principal components (PCs) of the entire peak lists of MALDI TOF MS mass spectra shows the distribution of the isolates of *M. abscessus (sensu stricto)* (blue), *M. massiliense* (red), and *M. bolletii* (green)

PCA using the entire peak lists clearly separated all *M. abscessus (sensu stricto)* and *M. massiliense* isolates into two distinct clusters, visible in the 3-dimensional subspace spun by the first three principal components (Fig. 1). However, two isolates of *M. abscessus (sensu stricto)* were located in the border region of the *M. massiliense* cluster. The *M. bolletii* isolates did not form a separate cluster but were localized in the *M. abscessus (sensu stricto)* cluster (Fig. 1).

**Fig. 2:**
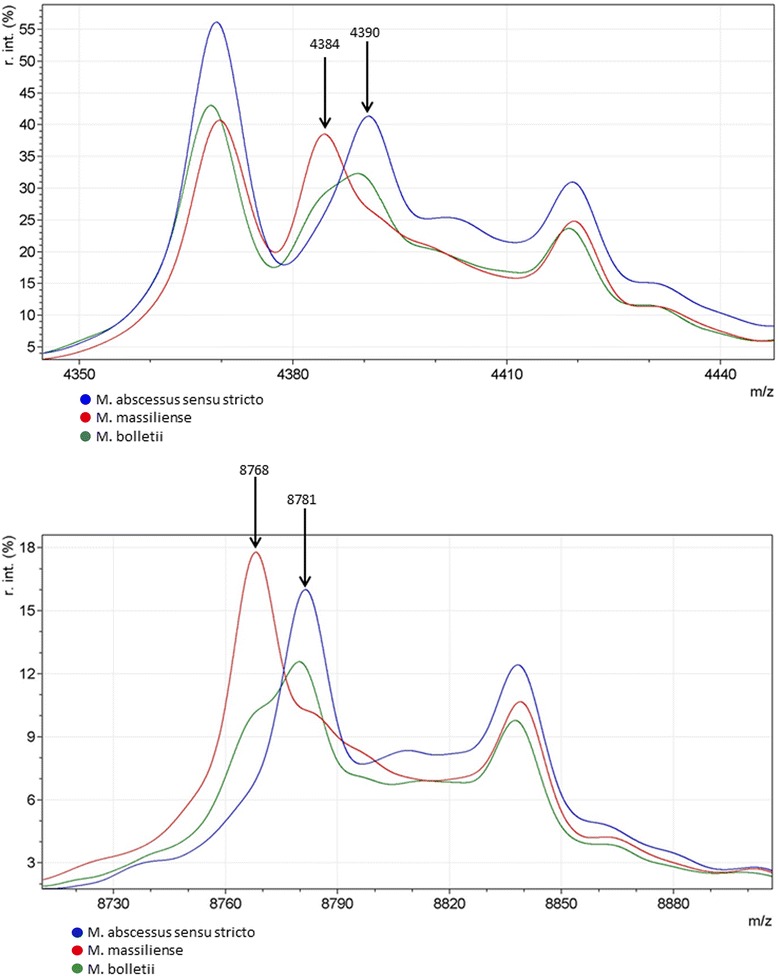
Matrix-assisted laser desorption/ionization time-of-flight (MALDI TOF) averaged spectral profiles of discriminating peak regions of *Mycobacterium abscessus* complex isolates. Diagram shows relative intensity (%) against mass-to-charge ratio (m/z) values of the discriminating peak regions of MALDI TOF averaged mass spectral profiles for *Mycobacterium abscessus (sensu stricto)* (blue), *M. massiliense* (red), and *M. bolletii* (green) isolates

Furthermore, we performed single peak analysis of all isolates to detect unique differences within the three subspecies. The spectra of *M. abscessus (sensu stricto)* isolates and *M. massiliense* isolates differed substantially in two areas. The averaged spectra demonstrate specific *M. massiliense* peaks at 4,384 and 8,768 m/z, whereas these peaks were missing from the *M. abscessus (sensu stricto)* and the *M. bolletii* averaged spectra (Fig. 2). Otherwise, the averaged spectra of *M. abscessus (sensu stricto)* and *M bolletii* isolates exhibited specific peaks at 4,390 and 8,781 m/z that were absent from the averaged spectrum of *M. massiliense* (Fig. 2).

With a relative peak intensity of 1 % as the threshold, single spectrum analysis demonstrated that the 4,384 m/z peak was prevalent in 89.5 % (17/19) of the *M. massiliense* isolates and the 8768 m/z peak was present in 57.9 % (11/19), whereas both peaks were absent from all *M. abscessus (sensu stricto)* and *M. bolletii* isolates (Table 1). The peak at 4,390 m/z was present in all *M. abscessus (sensu stricto)* isolates (28/28) and *M. bolletii* isolates (3/3), and the 8,781 m/z peak was present in 89.2 % (25/28) of *M. abscessus (sensu stricto)* isolates and 100 % of the *M. bolletii* isolates, whereas the 4,390 m/z peak was detected only in 5.2 % (1/19) of *M. massiliense* isolates and the 8,781 m/z peak was absent from all 19 *M. massiliense* isolates (Table 1). No specific peak for *M. bolletii* was detected by the peak-detection algorithm with a 1 % relative intensity threshold.

**Table 1:** Presence of the differentiating peaks in single spectra from matrix-assisted laser desorption/ionization time-of-flight mass spectrometry (MALDI TOF MS) among subspecies of *Mycobacterium abscessus* complex

Peak m/z	4384	4390	8768	8781
Subspecies	No (%)	No (%)	No (%)	No (%)
*Mycobacterium abscessus (sensu stricto), n* = 28	0 (0)	28 (100)	0 (0)	25 (89.3)
*Mycobacterium massiliense, n* = 19	17 (89.5)	1 (5.3)	11 (57.9)	0 (0)
*Mycobacterium bolletii, n* = 3	0 (0)	3 (100)	0 (0)	3 (100)

*m/z* mass-to-charge ratio

**Fig. 3:**
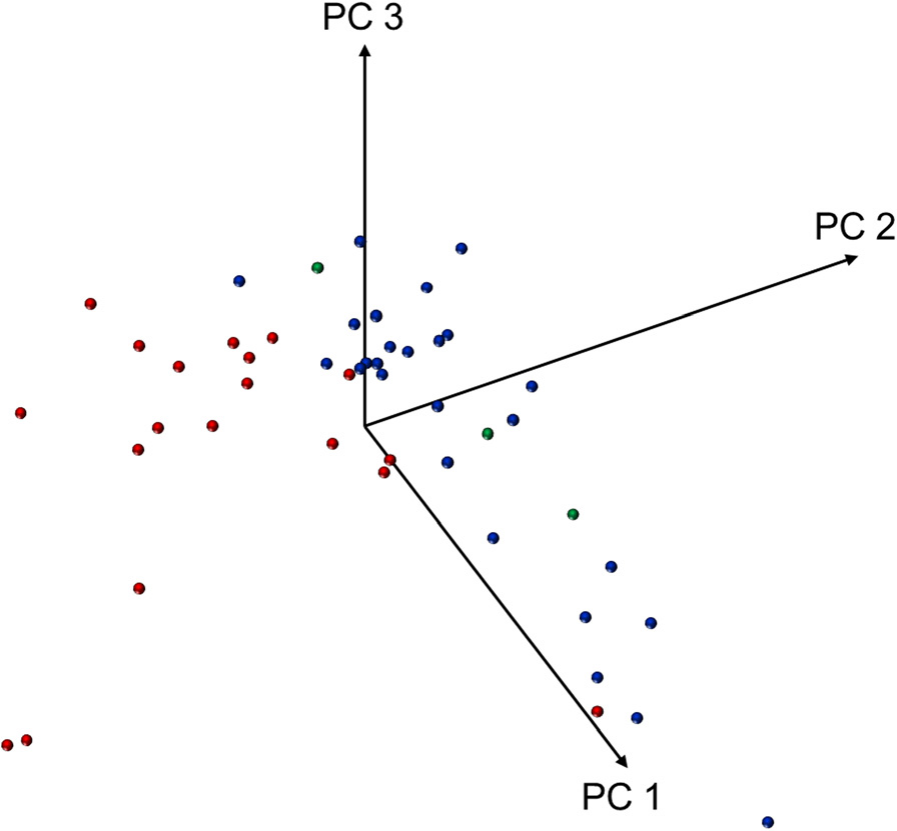
Principal component analysis of the matrix-assisted laser desorption/ionization time-of-flight mass spectrometry (MALDI TOF MS) peak lists of *M. abscessus* complex isolates excluding four discriminating peaks. Diagram of the first three principal components (PCs) of MALDI TOF MS peak lists excluding four discriminating peaks shows the distribution of the isolates *of Mycobacterium abscessus (sensu stricto)* (blue), *M. massiliense* (red), and *M. bolletii* (green)

**Fig. 4:**
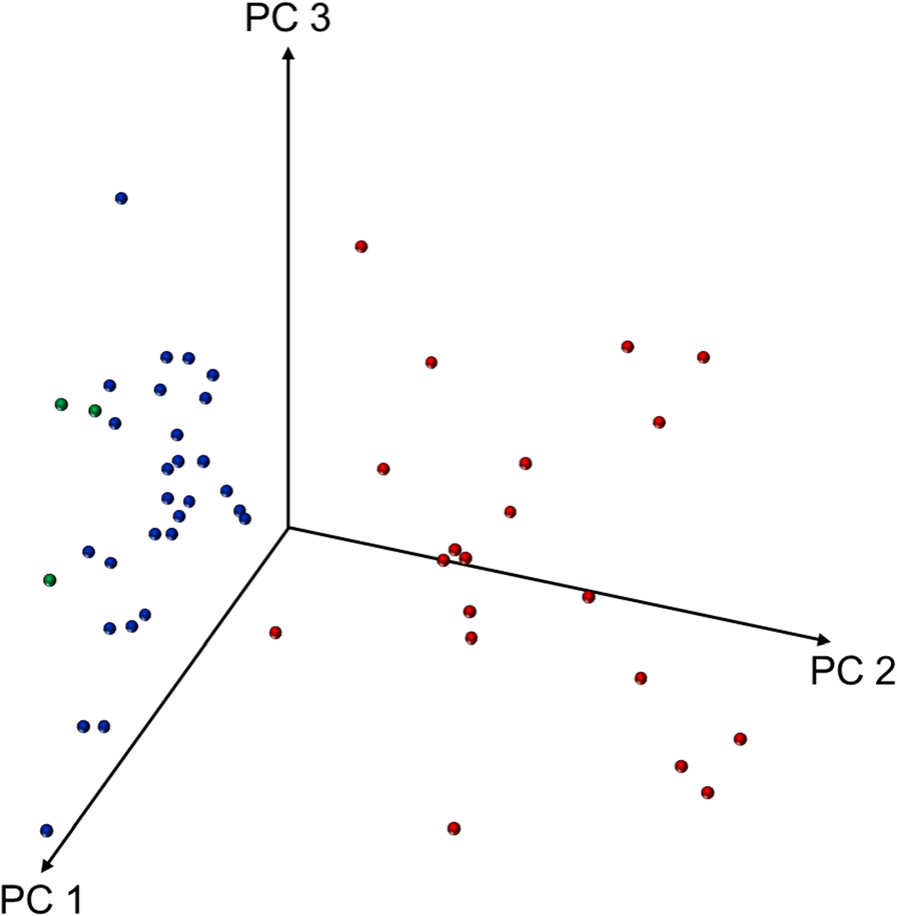
Principal component analysis of the *Mycobacterium abscessus* complex SuperSpectrum supplemented with discriminating peaks. Diagram of the first three principal components (PCs) of preset *M. abscessus* SuperSpectrum of SARAMIS 4.12 software, supplemented with discriminating peaks identified in our study, shows the distribution of the isolates of *M. abscessus (sensu stricto)* (blue), *M. massiliense* (red), and *M. bolletii* (green)

To analyze the relevance of the four discriminating peaks for separating *M. abscessus (sensu stricto)* isolates from *M. massiliense* isolates, we performed PCA by excluding these four peaks; nevertheless, we obtained a discrimination that was equally as good (Fig. 3) as that obtained with PCA including all peaks (Fig. 1). As Saramis 4.12 identifies *M. abscessus* at only the complex level, we examined whether a PCA restricted to the peaks included in the peak list of the *M. abscessus* SuperSpectra of the Saramis 4.12 database, supplemented with the discriminating peaks at 4,368 and 8,768 m/z, could distinguish *M. massiliense* and *M. abscessus (sensu stricto)*. This procedure indeed separated both subspecies in two clusters, whereas *M. bolletii* isolates were also represented within the *M. abscessus (sensu stricto)* cluster (Fig. 4).

## Discussion

In this study we examined the ability of VITEK MS Plus to differentiate the subspecies of the *M. abscessus* complex, comprising *M. abscessus (sensu stricto)*, *M. massiliense,* and *M. bolletii*, cultured in MGIT liquid medium. We used MALDI TOF MS peak lists for HCA and PCA; we also analyzed the peak lists relating to differences between unique subspecies. Although HCA did not reproduce a grouping according to the subspecies, PCA clearly distinguished *M. abscessus (sensu stricto)* isolates and *M. massiliense* isolates. The difference in the results obtained with the two analytical methods can be explained by the different underlying algorithms used for data analysis. HCA performs clustering by calculating Euclidean distances between the isolates, resulting in groups that exhibit multivariate similarity. In contrast, PCA finds those parameters that contribute to the maximal variance of the data and provide the best overall sample separation. In this way PCA reduces the number of dimensions for analysis and forms clusters that are based on inequalities [16, 17].

A previous study reported insufficient subspecies classification when entire peak lists were used for cluster analysis [18]. To the best of our knowledge, ours is the first study to use PCA as analytical method for discriminating the subspecies of the *M. abscessus* complex by MALDI TOF MS and to demonstrate that this analytical method is more useful than HCA for discriminating *M. abscessus (sensu stricto)* from *M. massiliense*. PCA from MALDI TOF MS peaks has been demonstrated to be an effective method of discriminating closely related bacterial or fungal species [19–22].

Comparison of average spectra demonstrated four peaks that were differentially present in discriminating *M. abscessus (sensu stricto)* from *M. massiliense*. It seems likely that these peaks form two pairs (4384/8768 and 4390/8781), in each case representing one molecule that is displayed twice in the peak spectrum, single charged on the one hand and double charged on the other. To date, three published studies have used single-peak analysis to discriminate MALDI TOF MS peaks for members of the *M. abscessus* complex [18, 23, 24]. In two of these studies only two subspecies were discriminated [18, 23], and only one study analyzed isolates of all three subspecies [24], including *M. bolletii*, which is the most rarely isolated subspecies. However, none of those peaks accurately discriminated all of the isolates included in that study [24]. The authors speculated that the degree of horizontal gene transfer that occurs in the evolution of *M. abscessus* may be responsible for the limitations in accuracy [24].

Furthermore, most of the discriminating peaks described so far differed between these three studies and could not be confirmed by the other studies. The only peak determined to be discriminating in two of the three previous studies [18, 23] was detected at approximately the 8,769 m/z value, which has been described as specific for *M. massiliense.* Our study also detected this specific peak, which showed 100 % specificity for discriminating the isolates included in our study. The other peaks were described as discriminative by only one of the three studies and were not found in the other two. Two of the three peaks that we found in our study have been described by Teng et al. as discriminative for *M. massiliense* and *M. abscessus (sensu stricto)* [23]. The description of only a few discriminating peaks by these published studies indicates that the MALDI TOF MS proteome is strongly related across the three subspecies. This finding is in line with the strongly related genotype of the three subspecies, which makes multilocus sequencing necessary for clear discrimination.

A specific reason for the inability of these studies to reproduce the discriminating MALDI TOF MS peaks is unclear so far. The extraction protocols do not appear to play an important role, because two studies, that by Teng et al. [23] and that by Fangous et al. [24], applied exactly the same protocols and used the same MALDI TOF MS device. It has been speculated that differences in the biogeographic MS profiles of *M. abscessus* complex subspecies may play a role [24]. Furthermore, peaks with a low relative intensity, which have also been described to be discriminative and appear below a relative intensity of 1 % [23, 24], may vanish after smoothing and baseline subtraction of the software, and therefore may not be confirmed by other studies. To avoid this issue, we considered only peaks with a relative intensity of at least 1 % as candidates for a discriminative peak.

Restricting the discrimination between the *M. abscessus* subspecies to only one or very few peaks that do not seem to be reproducible by other studies does not appear to be an appropriate method. The use of PCA is more appropriate in this case, because PCA that excludes the four discriminating peaks detected in our study delineates the subspecies equally as well as PCA that includes these peaks. This finding indicates that the importance of these single peaks for subspecies identification is low when this analytical method is used.

Our study is limited by the small number of isolates used: 28 isolates of *M. abscessus (sensu stricto)*, 19 isolates of *M. massiliense,* and 3 isolates of *M. bolletii,* which is rarely isolated. These small numbers may particularly influence discrimination with single-peak analysis, because bioregional variations may affect single MALDI TOF MS peaks more than they affect the entire peak spectrum.

## Conclusion

In this study, we showed that PCA of MALDI TOF MS peaks is a robust method that separates *M. massiliense* from the other subspecies of the *M. abscessus* complex. This finding is particularly relevant for clinicians, because *M. massiliense* exhibits a better response to antibiotic treatment [9]. Supplementing the VITEK MS Plus identification system with a PCA-based, trained support vector machine or using linear discriminant analysis of the existing peak lists of *M. abscessus* complex in the Saramis 4.12 database, including the discriminating peaks found by our study, could help to distinguish *M. massiliense* isolates in routine microbiological diagnostics.

## Abbreviations

c.l.: confidence level
CF: cystic fibrosis
CHCA: α-cyano-4-hydroxy cinnamic acid
HCA: hierarchical cluster analysis
ITS: internal transcribed spacer
IVD: in vitro diagnostic
m/z: mass-to-charge ratio
MALDI-TOF MS: matrix-assisted laser desorption/ionization time-of-flight mass spectrometry
MGIT: mycobacterium growth indicator tube
NTM: nontuberculous mycobacterium
PCA: principal component analysis
RUO: research use only
